# Early Naso-Gastric Feeding and Outcomes of Anorexia Nervosa Patients

**DOI:** 10.3390/nu15030490

**Published:** 2023-01-17

**Authors:** Maria Rosaria Marchili, Antonella Diamanti, Valeria Zanna, Giulia Spina, Cristina Mascolo, Marco Roversi, Benedetta Guarnieri, Gianluca Mirra, Giulia Testa, Umberto Raucci, Antonino Reale, Alberto Villani

**Affiliations:** 1 Department of Emergency, Acceptance and General Pediatrics, Bambino Gesù Children’s Hospital, IRCCS, 00165 Rome, Italy; 2 Hepatology, Gastroenterology and Nutrition Unit, Bambino Gesù Children’s Hospital, IRCCS, 00165 Rome, Italy; 3 Anorexia Nervosa and Eating Disorders Unit, Child Neuropsychiatry, Department of Neuroscience, Bambino Gesù Children’s Hospital, IRCCS, 00165 Rome, Italy; 4 University Hospital Pediatric Department, Bambino Gesù Children’s Hospital, IRCCS, University of Rome Tor Vergata, 00133 Rome, Italy; 5 Systems Medicine Department, University of Rome Tor Vergata, 00133 Rome, Italy

**Keywords:** anorexia nervosa, enteral nutrition, eating disorders, children

## Abstract

Nutritional rehabilitation with weight restoration is an important step in patients hospitalised for anorexia nervosa (AN). Naso-gastric feeding (NGF) should be considered when oral nutrition (OF) and oral nutritional supplementation (ONS) are insufficient. We evaluated the role of NGF on short- and long-term outcomes, considering weight gain, the length of hospitalisation (LOS) and the time to relapse. We report on the characteristics of patients under 18 years of age with AN admitted to the Department of Emergency and Acceptance of the Bambino Gesù Children’s Hospital, IRCCS, Rome, between March 2019 and August 2022. Three hundred and fifteen patients were enrolled. We compared patients treated with NGF (group A) and patients without NGF (group B). Group A was characterised by a significantly lower BMI on admission and discharge, more frequent use of inpatient psychotropic therapy (IPDT) and longer hospital stay. The time to relapse was significantly longer in group A compared to group B. An early NGF setting correlates with a longer time to relapse and may be associated with a shorter LOS. A high caloric intake with a balanced nutritional formula provided by NGF allows an earlier recovery. The main advantages of this approach could be the rapid discharge of patients and a more effective psychological and social recovery.

## 1. Introduction

Anorexia nervosa (AN) is one of the most frequent emerging psychiatric illnesses among young people and is related to high mortality rates, especially in patients with medical complications or psychiatric comorbidities [1,2,3,4]. It is characterised by a chronic course, high relapse rates and costs that represent a severe burden for both patients and families [5,6]. Due to its complexity, multidisciplinary management is necessary to achieve the best recovery for each patient [7,8]. To this end, nutritional rehabilitation with adequate weight restoration is an important first step for medical stabilisation and the prevention of short- and long-term complications in patients with AN [9]. Specific criteria for refeeding strategies have not yet been established [10] but most reports suggest a gradual introduction of food in order to avoid refeeding syndrome [11]. In patients with AN who refuse oral nutrition (OF), several artificial re-nutrition strategies have been described, such as oral nutritional supplements (ONS) if the total daily nutritional intake is not guaranteed, including drinking products with a normoproteic gluten-free composition and naso-gastric feeding (NGF) in the presence of any form of nutritional refusal without achieving the nutritional goal; rarely, parenteral nutrition (NP) is considered in patients with digestive insufficiency and life-threatening malnutrition [12]. Although there are currently no guidelines on the timing of weight restoration, the literature recommends NGF when the planned OF and ONS are insufficient or in cases of inadequate patient compliance [13]. NGF steadily improves weight gain, but no evidence on long-term outcomes is available [14]. Furthermore, the literature lacks guidance on the most appropriate mode of NGF administration (bolus, overnight regimen or continuous). A direct comparison between these three different regimens has not yet been performed [15].

In view of the lack of unambiguous evidence on the nutritional assessment of patients with AN, the aim of the present study was to focus on the management of patients admitted to the paediatric ward. In particular, we assessed the role of NGF in short- and long-term outcomes, considering aspects such as weight gain, the length of hospitalisation (LOS) and the time to relapse.

## 2. Materials and Methods

### 2.1. Study Design

In this study, we retrospectively reviewed the electronic medical records of all patients under 18 years of age admitted to the Department of Emergency and Acceptance of the Bambino Gesù Children’s Hospital, IRCCS, Rome, Italy, between March 2019 and August 2022, with a diagnosis of AN and admitted due to severe general and nutritional status. The Diagnostic and Statistical Manual of Mental Disorders fifth edition (DSM-5) was used for the diagnosis of AN [16].

Patients were excluded if no data on their weight status were reported, due to a lack of computerised records. We reported the following clinical and laboratory data: age (years); sex; residence in the Lazio region (where the admitting hospital is located) or elsewhere; admission before the COVID-19 pandemic; emergency triage coding (blue or higher); weight loss at admission (kg); time of weight loss before admission (months); Body Mass Index (BMI) at admission (kg/m^2^ and age-adjusted percentile); degree of thinness as defined by Cole [17]; duration of admission (days); BMI at discharge (kg/m^2^ and age-adjusted percentile); follow-up time (months); number of first or second relapses; time before first or second relapse (days); menarche; amenorrhoea; time to amenorrhoea (months); presence of any psychiatric disorder, including suicidal ideation or depression; presence of pericardial effusion; presence of any comorbidity; vital signs, including heart rate (bpm), systolic and diastolic blood pressure (mmHg) respiratory rate (rpm); presence of bradycardia; haemogasanalysis parameters, including pH, base excess and lactate (mmol/L); laboratory tests, including azotemia (mg/dL), creatinine (mg/dL), haemoglobin (g/dL), albumin (g/dL); serum assays of vitamin A (μM/mL), BN1 (ng/mL), B6 (ng/mL), B12 (pg/mL) and folic acid (ng/mL), vitamin C (μM/L), D (ng/mL) and E (μM/mL); serum dosages of hormones, including TSH (μIU/mL), FT4 (ng/dL), prolactin (ng/mL), FSH (ng/mL), LH (mIU/L), cortisol (μg/dL), ACTH (pg/mL), β-estradiol (ng/mL), progesterone (ng/mL) and testosterone (ng/dL); intravenous fluid administration; NGF administration, including the time before the start of nutrition, the duration of NGF (days) and whether it was administered overnight, during the day or all day; psychotropic therapy administration prior to admission; inpatient psychotropic therapy administration (IPDT), including the most commonly prescribed molecules.

A specialised team consisting of paediatricians, psychiatrists, psychologists, gastroenterologists, dietitians and nutrition nurses, was involved in the patient’s care following an institutional hospital protocol (IHP) [12] based on the National Institute for Health and Care Excellence guidelines on eating disorders [18]. The IHP defined the following admission criteria: BMI below the 3rd percentile according to age; bradycardia (heart rate < 50 bpm); QTc interval > 450 ms; hypothermia (axillary temperature < 35.5 °C).

In the case of low OF intake, according to the IHP, artificial nutrition should be considered. Specifically, if the caloric intake was less than 70%, OF was supplemented (ONS), whereas if the total caloric intake using both OF and ONS did not reach 30%, NGF was recommended. Both the ONS and NGF used normoprotein, with a caloric density of 1 kcal/mL or 1.5 kcal/mL, respectively; the protocols were gluten-free and with balanced macro- and micronutrient characteristics. Finally, we described a correlation between the treatments administered, i.e., ONS and IPDT, and the increase in BMI between admission and discharge, as well as LOS.

### 2.2. Statistical Analysis

The clinical and laboratory characteristics of all included patients were reported according to their statistical distribution. The patients were divided into two subgroups and compared according to the presence or absence of NGF. The software IBM SPSS version 23.0 was used for statistical analysis. Continuous normally distributed variables were presented as means ± standard deviations and analysed with the Mann–Whitney U-test. Categorical variables were expressed as proportions and percentages and analysed with the chi-square test or Fisher’s exact test (when appropriate). A *p*-value of less than 0.05 was considered statistically significant. Clinically and statistically significant associations from the bivariate analyses were included in the multivariate analysis, performed via logistic regression. Finally, we tested the association of NGF and IPDT with the differences in the mean percentile of BMI between admission and discharge and days of hospitalisation.

The Ethics Committee of the Bambino Gesù Children’s Hospital approved this study on the basis of the Declaration of Helsinki (revised in Seoul, Republic of Korea, October 2008).

## 3. Results

During the four-year study period, three hundred and fifteen patients were enrolled.

The detailed demographic and clinical characteristics of the study sample are shown in Table 1.

The mean age was 14.4 (SD 1.2) years. The majority of patients were female (n = 281, 89.2%). The mean BMI on admission was 15.5 kg/m^2^ (SD 2.6) and the median BMI percentile on admission was less than 3 (0.7; range 0.1–55). The median weight loss was 11.4 (SD 7.7) kg, with a median time to weight loss before admission of 4 months (IQR 6). The majority of patients had a leanness grade of 2 (22.2%) or 3 (43.2%), as defined by Cole [16]. The mean length of stay was 21 (SD 12) days. The mean BMI at discharge was 16.3 kg/m^2^ (SD 2.6), with a median percentile of BMI at discharge below 3 (2.5; IQR 16.8). Approximately 14.9% (n = 47) of patients relapsed during the study period, with a median time to relapse of 3.6 months.

All vital signs and laboratory tests, including vitamin assays, were within the normal range for age, except for a slight decrease in vitamin D with a mean of 26.9 ng/mL, just below the lower limit of normality of 30 ng/mL. The hormone assays were also normal, with only a modest increase, although within normal limits, in prolactin (mean of 14 ng/mL for a normal range of 4–15 ng/mL) and cortisol (mean of 16.4 μg/dL for a normal range of 6.0–18.4 μg/dL).

In our sample, 18.1% of the patients were diagnosed with a psychiatric disorder other than eating disorders and 60.2% of the study sample required the administration of psychotropic drugs, mainly aripiprazole (68.3%) and sertraline (44.8%).

Furthermore, inpatient management included intravenous fluid administration in the majority of the population (82.9%); 101 patients (32.1%) were supported with NGF with a median duration of NGF of (21 ± 13) days. In addition, ONS was performed in the entire study population.

**Figure 1:**
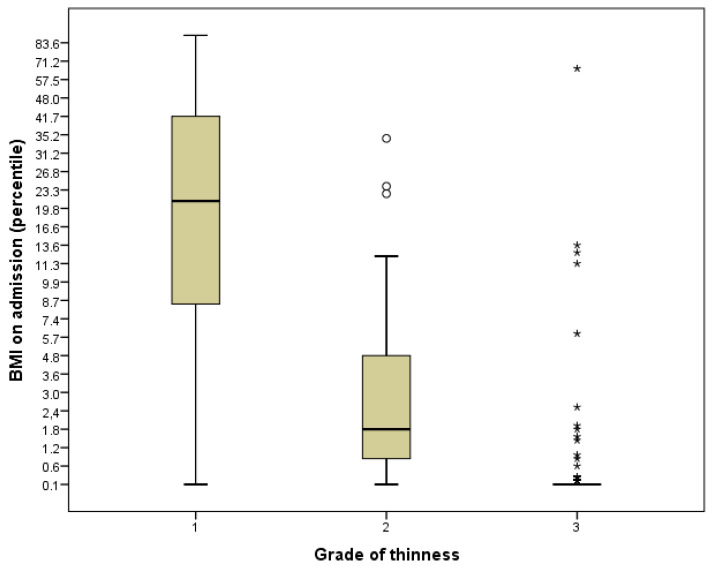
Boxplot of BMI on admission (percentile) stratified by grade of thinness. Hollow circles are outliers (more than 1.5 × IQR from the first and third quartile) and asterisks are extreme values (more than 3.0 × IQR from the first and third quartile).

As illustrated in Figure 1, we found a good correlation with the BMI percentile at the time of admission and the assigned degree of thinness, especially for grades 1 and 2.

We focused our research on nutrition management in patients with AN by dividing our population into two groups: patients treated with NGF (group A) and patients without NGF (group B).

Group A was characterised by significantly lower BMI percentiles at admission and discharge (*p* < 0.001). A longer LOS was also documented in group A, with a mean of 30 (SD 11) days compared to 16 (SD 9) days in group B (*p* < 0.001).

Psychotropic drug therapy was also used more frequently in group A than in group B (98 vs. 69.6%, *p* = 0.001). More comorbidities (47.5 vs. 35.5%, *p* = 0.042) and pericardial effusion cases (10.9 vs. 3.7%, *p* = 0.013) were found in group A than in group B.

The time to recurrence (months) was significantly longer in NGF-treated patients than in group B (median ± IQR: 8.2 ± 9.7 vs. 3.0 ± 6.0) (*p* = 0.035).

The full comparison between these two groups is summarised in Table 2.

By adjusting the clinically and statistically significant variables for each other in the multivariate analysis, as shown in Table 3, we confirmed that the time to relapse (in months) was significantly longer in patients who had been administered NGF than in untreated patients (OR 1.23, *p* = 0.050). Note that we did not include IPDT among the independent variables due to the occurrence of almost complete separation.

**Figure 2:**
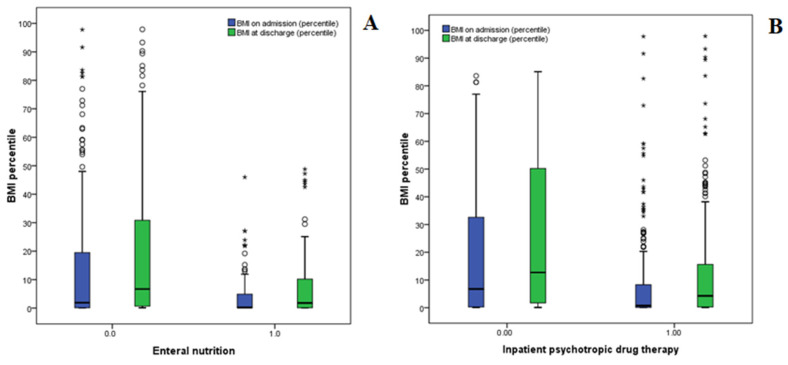
Effects of NGF (**A**) and IPDT (**B**) on BMI percentile increases. Hollow circles are outliers (more than 1.5 × IQR from the first and third quartile) and asterisks are extreme values (more than 3.0 × IQR from the first and third quartile).

Finally, we found correlations between NGF, IPDT and the increase in BMI between admission and discharge, as well as the LOS. As shown in Table 4 and Figure 2A,B, although significant percentile increases in BMI between admission and discharge were found in both groups (NGF and IPDT), no significant difference was found between the two groups. In other words, the EN and IPDT had similar impacts on the increase in BMI. However, the correlation between these two variables was not high, as shown by the calculation of a Pearson’s R value of just 0.324.

As shown in Table 5, NGF administration was significantly associated with longer a LOS (31 vs. 16 median days, *p* < 0.001).

**Figure 3:**
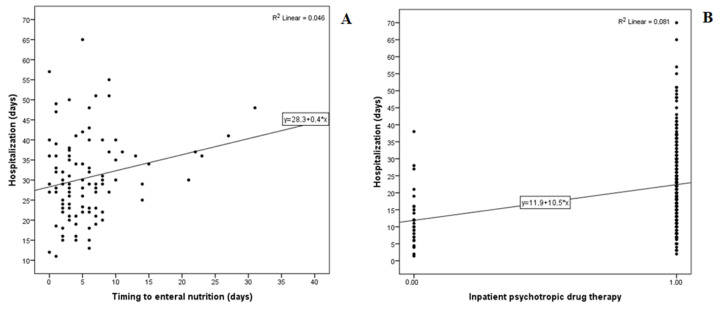
Effect of timing of NGF (**A**) and the administration of IPDT (**B**) on days of hospitalisation.

Therefore, we examined possible associations between the timing of the NGF administration and the LOS. As shown in Figure 3A, we found a positive correlation between these two variables, especially when NGF was administered after a hospitalisation period of 10 or more days. Similarly, IPDT was significantly associated with the LOS (22 vs. 12 median days, *p* < 0.001), as shown in Table 5 and Figure 3B.

## 4. Discussion

Although the hospital setting of NGF and the specific criteria are still debatable and controversial, weight restoration and nutritional recovery are still the key aspects of AN management [9,13]. Our study showed that 32.1% of hospitalised patients with AN required NGF due to a severe reduction in total caloric intake, which did not reach 30%. In particular, we found that patients with NGF had a more severe clinical picture, with a lower BMI, a higher rate of IPDT use and the presence of more comorbidities, such as pericardial effusion, than patients without NGF. In addition, patients requiring NGF had a longer LOS (mean ±SD: 30 ± 11 vs. 16 ± 9) (Table 2).

The impact of NGF on the LOS has been widely discussed in the literature. Similar to our results, some European studies have shown a longer LOS in NGF-treated patients [18,19,20]. In particular, a German retrospective study documented a longer LOS in NGF-treated patients, with mean LOSs of 136 and 82 days (*p* < 0.001), respectively [19]; a French retrospective study described a mean LOS of 180 days in the NGF group compared to 117 days in patients without NGF, concluding that NGF is a predictor of a longer LOS [20]. Similarly, Maginot and colleagues documented a longer LOS in patients treated with NGF, but these results included patients with more severe medical complications [21]. In contrast to our results, some researchers have found a shorter LOS in NGF-treated patients [21,22,23,24,25]. In particular, one study showed that OF requires a longer period for weight restoration due to the lower caloric component (50.9 days in cases of OF vs. 33.8 days in cases of NGF) [22]; Golden et al. showed that the LOS was significantly shorter in the NGF group (13.0 ± 7.3 days vs. 16.6 ± 9.0 days; *p* < 0.0001) [23]; faster weight restoration was described in a prospective study by Garber et al. [15,24,25].

The most likely reason for these differences from our results may be related to the private and more expensive healthcare system in the United States, where most of these studies were conducted. In contrast, a more conservative approach was adopted in our sample, especially in the first few days of hospitalisation, in addition to public healthcare management. Indeed, we documented that our longer LOS could be correlated with a latency of ten or more days before NGF administration; otherwise, timely NGF administration could be associated with a shorter LOS. The importance of reducing the LOS is related to both the total cost of hospitalisation and the impact on the national health system, as well as the social aspects involved in the patient’s return home (school, friends, family, etc.), in agreement with previous reports [26]. Recent research comparing inpatient treatment with outpatient treatment has described the advantages of this outpatient management approach; the rapid return to normal social activities and family environment favours a better treatment process, both for weight restoration and psychological recovery [27,28].

In addition, some Reports suggest discharging patients still receiving NGF in order to reduce the hospitalisation cost [29]. On the other hand, according to our pediatric experience, considering the risk associated with NGF management, home NGF has been proposed only in selected cases, for example for patients discharged in residential facilities with a dedicated medical team.

We found that patients requiring NGF had a longer remission period than patients without NGF support, with a mean time to relapse of 8.2 months compared to 3 months. This aspect highlights the importance of NGF on nutritional recovery, considering both qualitative and quantitative nutrients, exactly characterised as the caloric intake of micro and macronutrients. For the rest, our experience has shown that tube placement and NGF are directly correlated with increased patient compliance with OF and improved psychological picture. Consequently, we observed that patients treated with NGF have a longer total time to relapse after discharge; moreover, according to our IHP, these patients are strictly evaluated with a day hospital regimen [28]. This IHP allows patients and families rapid access to day hospital treatments, which allows them to focus on relational and psychological support after acute hospital management, ensuring both weight recovery and nutritional stabilisation.

With regard to pharmacological treatments, our data showed that NGF and IPDT have similar impacts on increasing BMI. Given the lack of clear standardisation of pharmacological treatment and paediatric-specific clinical studies, we highlighted the important role of NGF in the treatment of AN, especially in a paediatric population without psychiatric comorbidities. Moreover, in our experience, the wide range of side effects of common drugs used for the disease, the frequent hostility of patients and family members to start drug treatment and the poor compliance sometimes observed could be elements supporting the NGF setting in young patients with AN [6,30,31].

The future perspectives aim to provide standardised criteria for the setting and duration of NGF and for the modalities (bolus, overnight or continuous regimen). Furthermore, considering the duration of hospitalisation of patients with AN, future studies should assess the economic impacts of the hospital management of AN in order to optimise the use of therapeutic resources.

Our study provides useful information on the use of NGF by focusing on a large and homogeneous sample. The presence of IHP management and a multi-specialist team is an important aspect of the treatment of patients with AN. The follow-up data after discharge can be considered a strength of our research.The main limitation of this study is that it is a retrospective study subject to systematic bias; moreover, all the patients included received a psychiatric, psychological and nutritional evaluation belonging to a third-level inpatient treatment regimen. For this reason, our results and conclusions should be wisely compared and generalised to patients treated in different settings. In addition, a limitation of our study is the heterogeneity of the sample considering the different ages of the patients studied, from late childhood to adolescence.

## 5. Conclusions

This study shows that nutritional treatment is a key step in the recovery of patients, both for immediate and late consequences. In particular, an early NGF setting correlates with a shorter duration of hospitalisation and a longer time to relapse. A high caloric intake with a balanced micro- and macronutrient formula provided via NGF allows earlier weight restoration and recovery of the clinical picture. The main advantage of this approach could be the rapid discharge of patients, and consequently more effective psychological, emotional and social recovery.

## Figures and Tables

**Table 1:** Clinical characteristics of study sample.

Total	315	N/A
Age (years)—mean ± SD (range)	14.4 ± 1.2 (5.8–17.9)	0
Females—no. (%)	281 (89.2)	0
Before COVID-19 pandemic—no. (%)	77 (24.4)	0
Triage coding (blue or higher)—no. (%)	245 (77.8)	29
Weight loss on admission (kg)—mean ± SD (range)	11.4 ± 7.7 (0.5–40.0)	46
Weight loss to admission (months)—median ± IQR (5°–95°)	4 ± 6 (0.7–12.0)	40
BMI on admission (value)—mean ± SD (range)	15.5 ± 2.6 (9.0–32.0)	1
BMI on admission (percentile)—median ± IQR (5°–95°)	0.7 ± 8.7 (0.1–55.0)	1
Grade of thinness—no. (%) - Grade 1 - Grade 2 - Grade 3	109 (34.6) 70 (22.2) 136 (43.2)	0 0 0
LOS (days)—mean ± SD (range)	21 ± 12 (1–70)	39
BMI at discharge (value)—mean ± SD (range)	16.3 ± 2.6 (11.9–32.3)	27
BMI at discharge (percentile)—median ± IQR (5°–95°)	2.5 ± 16.8 (0.1–56.2)	27
Follow-up (months)—median ± IQR (5°–95°)	3 ± 5 (1–12)	-
Relapse—no. (%) Time to relapse (months)—median ± IQR (5°–95°)	47 (14.9) 3.6 ± 8.0 (0.4–14.9)	- -
2° relapse—no. (%) Time to 2° relapse (months)—median ± IQR (5°–95°)	6 (1.9) 5.0 ± 4.0 (0.6–12.9)	- -
Menarche—no. (%)	177 (56.2)	-
Amenorrhea—no. (%) Amenorrhea (months)—median ± IQR (5°–95°)	164 (52.1) 5.4 ± 5.6 (0.3–16.2)	- -
Psychiatric disorder *—no. (%)	57 (18.1)	-
Suicidal ideation—no. (%)	16 (5.1)	-
Depression—no. (%)	26 (8.3)	-
Pericardial effusion—no. (%)	19 (6.0)	-
Any comorbidity—no. (%)	124 (39.4)	-
**Vital Signs**		
Heart rate (bpm)—mean ± SD (range)	63.7 ± 18.2 (33–128)	14
Bradycardic—no. (%)	104 (33.0)	-
Systolic Blood Pressure (mmHg)—mean ± SD (range)	104 ± 12 (69–167)	27
Diastolic Blood Pressure (mmHg)—mean ± SD (range)	66 ± 9 (39–94)	27
Respiratory Rate (rpm)—mean ± SD (range)	17 ± 3 (12–28)	79
**Blood Gas Analysis**		
pH (value)—mean ± SD (range)	7.36 ± 0.05 (7.20–7.52)	127
Base Excess (value)—mean ± SD (range)	1.6 ± 4 (−15–13)	130
Lactate (mmol/L)—mean ± SD (range)	1.2 ± 0.5 (0.2–3.7)	214
**Laboratory workup**		
Azotemia (mg/dL)—mean ± SD (range)	13 ± 5 (2–40)	17
Creatinine (mg/dL)—mean ± SD (range)	0.7 ± 0.3 (0.03–1.4)	16
Hb (g/dL)—mean ± SD (range)	13.5 ± 1.1 (9.1–16.7)	16
Albumin (g/dL)—mean ± SD (range)	4.8 ± 0.4 (3.8–6.0)	46
Vitamin A (0.7–2.8 μM/mL)—mean ± SD (range)	1.4 ± 0.7 (0.4–4.1)	156
Vitamin B1 (32–95 ng/mL)—mean ± SD (range)	60.1 ± 36.0 (5.0–289)	149
Vitamin B6 (8.7–27.2 ng/mL)—mean ± SD (range)	36.2 ± 26.5 (5.2–203)	148
Vitamin B12 (197–711 pg/mL)—mean ± SD (range)	739 ± 422 (4–3100)	106
Folic acid (5–27.2 ng/mL)—mean ± SD (range)	8.4 ± 5.5 (1.8–40.3)	127
Vitamin C (26.1–84.6 μM/L)—mean ± SD (range)	55.0 ± 46.5 (0.3–349)	126
Vitamin D (30–100 ng/mL)—mean ± SD (range)	26.9 ± 8.1 (5.3–54.8)	98
Vitamin E (12.7–39.4 μM/mL)—mean ± SD (range)	28.0 ± 26.5 (9.0–310)	178
TSH (0.51–4.30 μIU/mL)—mean ± SD (range)	2.20 ± 1.16 (0.06–6.70)	76
FT4 (0.98–1.64 ng/dL)—mean ± SD (range)	1.13 ± 0.23 (0.57–2.11)	77
PRL (4–15 ng/mL)—mean ± SD (range)	14 ± 13 (0.6–74.1)	182
FSH (1.5–8.9 ng/mL)—mean ± SD (range)	3.2 ± 3.2 (0.3–18.1)	228
LH (0.7–17.8 mIU/L)—mean ± SD (range)	2.0 ± 6.7 (0.3–66.8)	200
Cortisol (6.0–18.4 μg/dL)—mean ± SD (range)	16.4 ± 5.2 (3.7–27.3)	222
ACTH (7.3–63.3 pg/mL)—mean ± SD (range)	23.5 ± 38.9 (2.3–357)	228
β-estradiol (12.4–398 ng/mL)—mean ± SD (range)	17.5 ± 36.0 (5.00–325)	215
Progesterone (0.05–14.5 ng/mL)—mean ± SD (range)	0.37 ± 1.07 (0.05–9.31)	240
Testosterone (4.6–38.3 ng/dL)—mean ± SD (range)	61.6 ± 126 (5.4–440)	304
**Management**		
Intravenous fluids—no. (%)	261 (82.9)	-
NGF—no. (%) Timing to NGF—median ± IQR (5°–95°) NGF duration—median ± IQR (5°–95°) NGF overnight—no. (%) NGF during daytime– no. (%) NGF all day—no. (%)	101 (32.1) 5 ± 5 (0–17) 21 ± 13 (9–44) 60 (19.0) 26 (8.3) 2 (0.6)	- - - - -
Outpatient psychotropic drug therapy—no. (%)	73 (23.2)	-
Inpatient psychotropic drug therapy—no. (%)	254 (80.6)	-
Aripiprazole—no. (%)	215 (68.3)	-
Sertraline—no. (%)	141 (44.8)	-
Fluoxetine—no. (%)	29 (9.2)	-
Diazepam—no. (%)	19 (6.0)	-
Other—no. (%) **	49 (15.7)	-
Guarding—no. (%)	17 (5.4)	-
Brain MRI—no. (%)	134 (42.5)	-
Pathologic brain MRI—no. (%)	17 (5.4)	-

* Psychiatric disorder other than eating disorder. ** Olanzapine n = 15 (4.8%), delorazepam n = 15 (4.8%), alprazolam n = 13 (4.1%), risperidone n = 6 (1.9), amitriptyline n = 3 (1.0%), lithium n = 3 (1.0%).

**Table 2:** Comparison of patients treated and untreated with NGF *.

	NGF (Group A)	No NGF (Group B)	*p*-Value
**Total**	**101**	**214**	
Age (years)—mean ± SD (range)	14.6 ± 1.8 (9.1–17.7)	14.4 ± 2.3 (5.8–17.9)	0.369
Females—no. (%)	92 (91.1)	189 (88.3)	0.459
Weight loss on admission (kg)—mean ± SD (range)	11.3 ± 7.9 (1.2–32.5)	11.4 ± 7.6 (0.5–40.0)	0.934
Weight loss to admission (months)—median ± IQR (5°–95°)	6 ± 6 (1–21)	(0.5–12)	0.151
BMI on admission (value)—mean ± SD (range)	14.5 ± 1.9 (10.6–19.0)	16.1 ± 2.8 (9.0–32.0)	<0.001
BMI on admission (percentile)—median ± IQR (5°–95°)	0.2 ± 5.1 (0.1–22.1)	1.9 ± 19.5 (0.1–64.7)	<0.001
Grade of thinness—no. (%) Grade 1 Grade 2 Grade 3	27 (38.6) 16 (22.9) 27 (38.6)	82 (33.5) 54 (22.0) 109 (44.5)	0.429 0.885 0.378
LOS (days)—mean ± SD (range)	30 ± 11 (11–65)	16 ± 9 (1–70)	<0.001
BMI at discharge (value)—mean ± SD (range)	15.5 ± 1.7 (12.0—20.0)	16.7 ± 2.8 (12.0 -32.0)	<0.001
BMI at discharge (percentile)—median ± IQR (5°–95°)	1.8 ± 10 (0.1–43.7)	6.7 ± 31.0 (0.1–74.6)	0.001
Follow-up (months)—median ± IQR (5°–95°)	(1–14)	(1–12)	0.921
Relapse—no. (%) Time to relapse (months)—median ± IQR (5°–95°)	14 (13.9) 8.2 ± 9.7 (–)	33 (15.4) 3.0 ± 6.0 (–)	0.717 0.035
2° relapse—no. (%)	1 (1.0)	5 (2.3)	0.668
Menarche—no. (%)	61 (60.4)	119 (55.6)	0.423
Amenorrhea—no. (%) Amenorrhea (months)—median ± IQR (5°–95°)	56 (91.8) 6 ± 5 (0.7–38.5)	108 (90.8) 5 ± 5 (0.03–16)	0.815 0.049
Psychiatric disorder **—no. (%)	20 (19.8)	37 (17.3)	0.589
Suicidal ideation—no. (%)	9 (8.9)	7 (3.3)	0.033
Depression—no. (%)	10 (9.9)	16 (7.5)	0.466
Pericardial effusion—no. (%)	11 (10.9)	8 (3.7)	0.013
Any comorbidity—no. (%)	48 (47.5)	76 (35.5)	0.042
**Vital Signs**			
Heart rate (bpm)—mean ± SD (range)	61 ± 17 (33–109)	65 ± 19 (33–128)	0.053
Bradycardic—no. (%)	47 (46.5)	57 (26.6)	<0.001
**Blood Gas Analysis**			
pH (value)—mean ± SD (range)	7.36 ± 0.05 (7.24–7.50)	7.35 ± 0.04 (7.20–7.52)	0.084
Base Excess (value)—mean ± SD (range)	1.6 ± 4.6 (−13.0–13.3)	1.6 ± 3.8 (−15.0–13.0)	0.916
Lactate (mmol/L)—mean ± SD (range)	1.2 ± 0.6 (0.2–3.7)	1.2 ± 0.4 (0.4–2.4)	0.939
**Laboratory workup**			
Azotemia (mg/dL)—mean ± SD (range)	14 ± 6 (2–39)	13 ± 5 (4–40)	0.126
Creatinine (mg/dL)—mean ± SD (range)	0.7 ± 0.2 (0.04–1.3)	0.7 ± 0.3 (0.03–1.4)	0.039
Hb (g/dL)—mean ± SD (range)	13.5 ± 1.1 (11.3–16.0)	13.5 ± 1.1 (9.1–16.7)	0.634
Albumin (g/dL)—mean ± SD (range)	4.8 ± 0.4 (3.8–5.8)	4.8 ± 0.4 (3.9–6.0)	0.075
**Management**			
Intravenous fluids	101 (100)	160 (74.8)	<0.001
Outpatient psychotropic drug therapy—no. (%)	27 (26.7)	46 (21.6)	0.314
Inpatient psychotropic drug therapy—no. (%)	99 (98.0)	149 (69.6)	<0.001
Aripiprazole—no. (%)	93 (92.1)	122 (57.0)	<0.001
Sertraline—no. (%)	63 (62.4)	78 (36.4)	<0.001
Fluoxetine—no. (%)	21 (20.8)	8 (3.7)	<0.001
Diazepam—no. (%)	9 (8.9)	10 (4.7)	0.140
Other—no. (%)	20 (19.8)	29 (13.6)	0.153
Guarding—no. (%)	10 (9.9)	7 (3.3)	0.016
Brain MRI—no. (%)	55 (54.5)	79 (36.9)	0.003
Pathologic brain MRI—no. (%)	5 (9.3)	12 (15.4)	0.302

** Psychiatric disorder other than eating disorder.

**Table 3:** Multivariate logistic regression (dependent variable: enteral nutrition administered).

Total	OR	C.I. 95%	*p*-Value
Before COVID-19 pandemic (yes)	1.891	0.285–12.561	0.510
BMI on admission (percentile)	0.870	0.736–1.029	0.104
Grade of thinness (value)	0.669	0.209–2.141	0.499
BMI at discharge (percentile)	1.031	0.973–1.093	0.299
Time to relapse (months)	1.198	1.015–1.415	0.033
Bradycardic (yes)	0.692	0.110–4.334	0.694

**Table 4:** Mean BMI percentile differences between admission and discharge (effects of NGF and IPDT).

		*p*-Value
All subjects—mean ± SD (range)	4.7 ± 12.0 (−31.2–92.4)	-
NGF—mean ± SD (range) No NGF—mean ± SD (range)	3.7 ± 7.8 (−6.8–48.7) 5.2 ± 13.6 (−31.2–92.4)	0.314
IPDT– mean ± SD (range) No IPDT—mean ± SD (range)	5.0 ± 11.7 (−23.3–92.4) 3.5 ± 13.1 (−31.2–56.3)	0.419

**Table 5:** Mean days of hospitalisation (effects of NGF and IPDT).

		*p*-Value
All subjects—mean ± SD (range)	21 ± 12 (1–70)	-
NGF–mean ± SD (range) No NGF—mean ± SD (range)	31 ± 11 (11–65) 16 ± 9 (1–70)	<0.001
IPDT—mean ± SD (range) No IPDT—mean ± SD (range)	22 ± 12 (2–70) 12 ± 8 (1—38)	<0.001

## Data Availability

Requests for access to data, statistical codes, questionnaires and technical processes may be made by contacting the corresponding author at giulia.spina@opbg.net.
